# The Effects of Unit Cell Arrangement and Hybrid Design on the Compressive Performances of Additive Manufactured Corrax Maraging Stainless Steel Lattices

**DOI:** 10.3390/ma18194443

**Published:** 2025-09-23

**Authors:** Ming-Hsiang Ku, Shou-Wun Chen, Cheng-Da Wu, Yan-Ting Liu, Quiao-En Lin, Chien-Lun Li, Ming-Wei Wu

**Affiliations:** 1 Department of Materials and Mineral Resources Engineering, National Taipei University of Technology, No. 1, Sec. 3, Zhong-Xiao E. Rd., Taipei 10608, Taiwan; lenno6622@ntut.edu.tw (M.-H.K.); kevinchen891217@gmail.com (S.-W.C.); a0956086876@gmail.com (Q.-E.L.); 2 Institute of Materials Science and Engineering, National Taipei University of Technology, No. 1, Sec. 3, Zhong-Xiao E. Rd., Taipei 10608, Taiwan; 3 Department of Mechanical Engineering, Chung Yuan Christian University, No. 200, Zhongbei Rd., Zhongli Dist., Taoyuan 32023, Taiwan; nanowu@cycu.edu.tw (C.-D.W.); yantingliu12@gmail.com (Y.-T.L.); 4 voestalpine Technology Institute (Asia) Co., Ltd., Rm. B105, No. 2, Wenxian Rd., Nantou 54041, Taiwan; allen.licl@voestalpine.com

**Keywords:** selective laser melting, compressive behavior, corrax maraging stainless steel, lattice structure, specific energy absorption

## Abstract

Selective laser melting (SLM) enables the production of complexly shaped metals with programmable mechanical responses, yet most lattice studies still rely on a single unit cell and a simple columnar build, severely restricting performance improvements. Here, we examine how combining distinct unit cells and rearranging them within the build volume affects the compressive behavior of SLM Corrax maraging stainless steel lattice structures. Three designs are additively manufactured as follows: a columnar cubic-FCCZ lattice, an alternating cubic and FCCZ lattice, and a hybrid lattice (cubic+FCCZ unit cell). In situ 2D digital image correlation (DIC) and finite element analysis (FEA) are used to resolve full-field strain evolution and failure modes under quasi-static compression. The hybrid lattice achieves the highest first maximum compressive strength (418 ± 5.78 MPa) and energy absorption (128.5 ± 6.83 MJ/m^3^), with its specific energy absorption (44.2 ± 1.48 kJ/kg) outperforming that of the columnar cubic-FCCZ lattice and alternating cubic and FCCZ lattice by 21.76% and 8.07%, respectively. The enhanced performance is attributed to the more uniform stress distribution and delayed shear band localization afforded by the hybrid lattice. These findings show that simultaneously optimizing unit cell geometry and arrangement can significantly expand the design space of metal lattices and provide a practical approach to improving the compressive strength and energy absorption capacity of load-bearing SLM components.

## 1. Introduction

Selective laser melting (SLM) is an additive manufacturing (AM) technology [1,2,3] capable of fabricating metal components with complex geometries, such as periodic or stochastic lattice structures. SLM is widely applied in the production of metallic materials, including iron-based alloys [4,5,6,7], titanium-based alloys [8,9,10,11], aluminum-based alloys [12,13,14], and other types of alloys [15,16,17,18]. Lattice structures produced by SLM exhibit high specific strength and high specific energy absorption, which contribute to enhanced mechanical performance and lightweight design. As a result, SLM lattices have attracted significant attention and research across various engineering fields [19,20,21,22,23].

Corrax maraging stainless steel combines high strength, toughness, and corrosion resistance, making it valuable for tooling and structural applications. Additive manufacturing enables the formation of complex lattice structures that enhance lightweight design and energy absorption, which cannot be achieved by conventional methods. Therefore, this study addresses the practical task of fabricating Corrax lattice structures via SLM to develop advanced functional materials.

Unit cells are the fundamental and repeating elements in the design of SLM lattice structures. Various types of unit cells have been applied in the fabrication of SLM lattices, including cubic, face-centered cubic with a Z-axis strut (FCCZ), and triply periodic minimal surfaces (TPMS) [24,25,26,27]. In general, the mechanical properties of SLM lattice structures depend on the type of unit cell and its arrangement [28,29,30]. Therefore, increasing attention has been paid to studies on the influence of unit cell types and arrangement designs on lattice performance. Lee et al. [29] designed five different unit cell arrangement combinations to investigate the compressive performances and deformation behaviors of lattice structures. Their study showed that layered lattice structures exhibited a mixed deformation mode, with the Row-type configuration demonstrating the best absorption energy characteristics. Lin et al. [30] investigated the effects of two types of functionally graded materials (FGM) on the compressive performance and fracture mechanisms of SLM manufactured Corrax maraging stainless steel lattices. The results showed that the FGM-V lattice exhibited specific energy absorption at 50% strain that was 7.6% and 19.4% higher than that of the FCCZ and cubic structures, respectively. This improvement was attributed to the effective mitigation of strain localization, which delayed the onset of failure.

In addition to single unit cell structures and arrangement designs, some researchers have also investigated the effects of mixed unit cell designs on mechanical properties [31,32,33,34]. Rahimi et al. [31] proposed a checkerboard (FRB) structure composed of alternating FCC and body-centered cubic (BCC) cells, as well as a uniformly mixed (Hybrid) structure. The results indicated that the FRB structure was suitable for lightweight energy absorption applications, while the hybrid structure was more appropriate for high-load applications. Ma et al. [32] also designed a hybrid lattice combining octet cells (OCT) and BCC units, and their results showed that its crush force efficiency (CFE) was higher than that of the OCT structure, with a maximum value exceeding 0.9. Xiao et al. [33] studied LPBF 316L alloy and directly combined RD and OCT unit cells to obtain a new hybrid OR lattice. The hybrid unit cell exhibited higher initial strength than the RD unit cell, which contributed to its superior energy absorption capacity. In addition to structural design, improving the grain structure and reducing porosity through heat treatment [35,36] and hot isostatic pressing (HIP) [37,38] can also help improve the mechanical properties of SLM metal lattices.

Different lattice structures demonstrate distinct deformation behaviors and mechanical properties under compression, bending, and energy absorption. However, most current studies focus on single unit cell types and arrangements, limiting the overall design space [19,26,28,37]. This study explores how varying unit cell types and arrangement strategies affect the compressive performance of SLM Corrax lattice structures. Digital image correlation (DIC) and finite element analysis (FEA) were employed to analyze and validate the stress–strain distributions and fracture mechanisms during compression. Results showed that hybrid lattices composed of cubic and FCCZ unit cells had higher specific energy absorption (SEA) by 21.76% and 8.07%, respectively, compared to the columnar cubic-FCCZ lattice and alternating cubic and FCCZ lattice. Furthermore, alternating arrangements of cubic and FCCZ lattices increased SEA by 12.67% compared to columnar configurations. These findings suggest that using optimized unit cell combinations (e.g., a hybrid lattice) and arrangement strategies (e.g., alternating or uniform distribution) can significantly enhance the compressive strength and energy absorption capacity of SLM Corrax lattices.

## 2. Material and Methods

In this study, Corrax stainless steel powders with high sphericity were produced via gas atomization. The median particle size (D_50_) was 37 μm, as measured by laser light scattering particle analyzer (Mastersizer 3000E, Malvern Instruments LTD., Worcestershire, UK) [5]. The chemical composition is listed in Table 1. Lattice specimens were fabricated using SLM equipment (EOS M290, EOS GmbH, Krailling, Germany), with the build direction aligned along the Z-axis. The SLM parameters [5] are listed in Table 2. For each group, three to five specimens were fabricated and tested. Based on the literature [38], post-processing heat treatment was conducted to eliminate residual stress and anisotropy and to enhance the mechanical properties. Initially, the samples were heated to 850 °C at a rate of 10 °C/min and held for 0.5 h for solution treatment. Subsequent cooling to room temperature was carried out under a nitrogen atmosphere at 0.5 MPa. Aging treatment was then performed in an air furnace by reheating the samples to 525 °C at the same heating rate and holding for 4 h, followed by furnace cooling.

**Figure 1:**
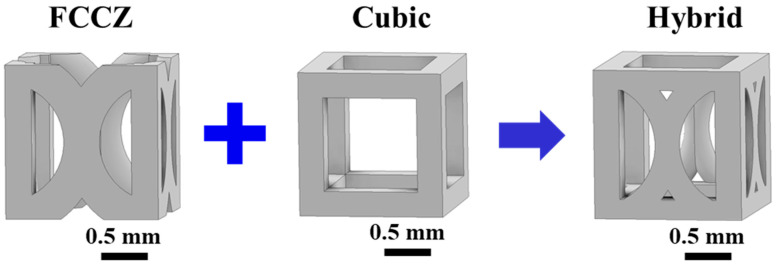
The unit cells adopted in this study.

**Figure 2:**
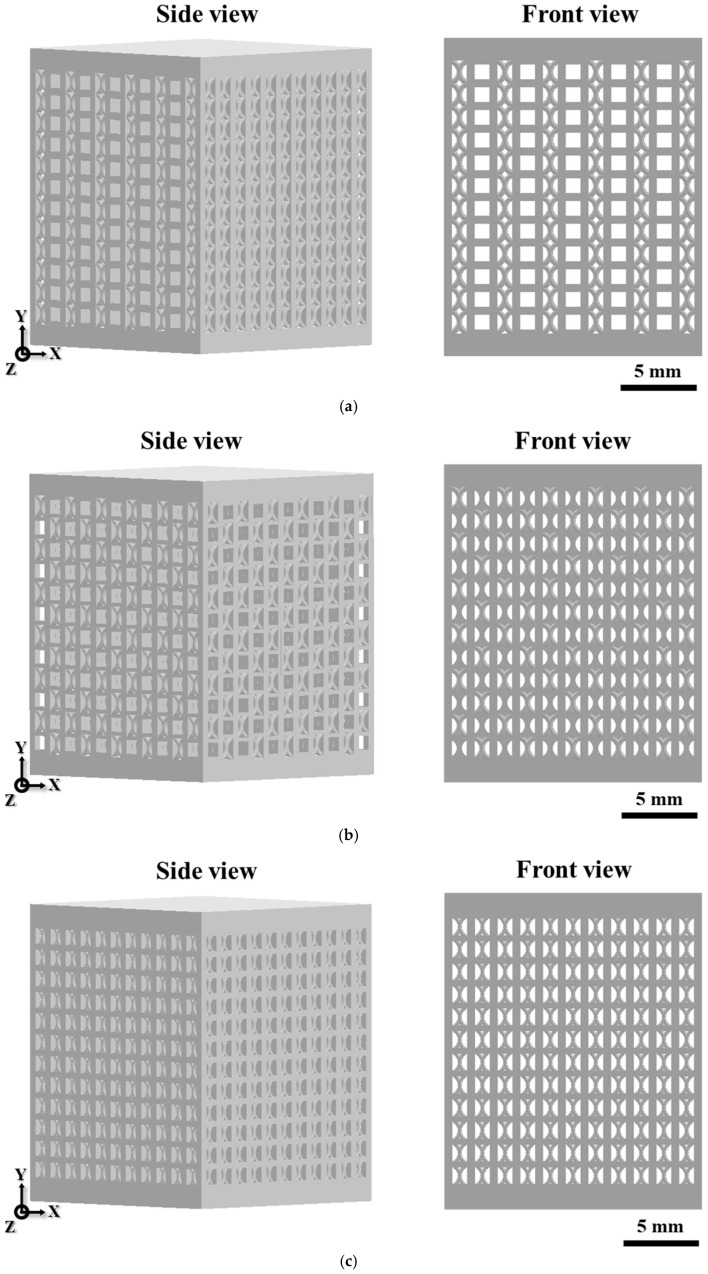
Schematics of SLM Corrax specimens: (**a**) C (**b**) A (**c**) H.

Three types of unit cell geometries were adopted for the lattice design: cubic, FCCZ, and a hybrid combination of the two (denoted as Hybrid), as shown in Figure 1. All unit cells were designed with dimensions of 1.5 × 1.5 × 1.5 mm^3^, corresponding to surface areas of 13.85 mm^2^, 15.9 mm^2^, and 17.26 mm^2^, respectively. The relative density of all the lattice structures was set to 0.3. Based on different combinations of unit cell geometries, three lattice designs—C, A, and H—were constructed, as illustrated in Figure 2. The C specimen was composed of cubic unit cells and FCCZ unit cells stacked into columns and arranged in sequence (Figure 2a), while the A specimen featured alternating arrangements of the two (Figure 2b). The H specimen was entirely composed of the hybrid (cubic+FCCZ) unit cell (Figure 2c). In the C and A designs, the cubic-to-FCCZ ratios were 5:6 and 1:1, respectively. All specimens were fabricated with top and bottom solid layers (1.5 mm thickness) to ensure stability during compression testing.

To evaluate the porosity and relative density of each lattice specimen, Archimedes’ method was employed. The measured density of each specimen was divided by the theoretical density of Corrax stainless steel to calculate the actual relative density and was tested three to five times. Surface morphology of the as-built lattice structures was examined by scanning electron microscopy (SEM) to assess the quality of the struts and identify potential surface defects. After the uniaxial compression tests, fracture locations and crack propagation paths were observed to determine the failure mechanisms. Special attention was given to how different unit cell arrangements influenced deformation behavior and structural failure modes.

To investigate the differences in mechanical performance among the three types of porous specimens after solution-aging (SA) treatment, uniaxial compression tests were performed using a universal testing machine (HT-9510, Hung Ta Instrument Co., Taichung, Taiwan), following the ISO 13314:2011 standard [39] for porous and cellular metals. Each specimen was compressed at an initial speed of 1.08 mm/min until a total strain of 50% was reached. For statistical reliability, each specimen type was tested three to five times. First maximum compressive strength and specific strength were recorded for analysis. Energy absorption (EA) and specific energy absorption (SEA) were calculated using Equations (1) and (2), as defined in Reference [39], where *e* (%) is the compressive strain, *e*_n_ (%) is the final compressive strain, *σ* (MPa) is the compressive stress, *σ*_n_ (MPa) is the final compressive stress, and ρth is the theoretical density of material. These parameters were substituted into the following equations to obtain the absorbed energy *W* (MJ/mm^3^) and specific energy absorption (*SEA*, kJ/kg):

(1) $$W=\frac{1}{100}\int_{0}^{e_{n}}\sigma \mathrm{de}\;\approx \;\frac{1}{100}[\frac{\left(e_{2}-e_{1}\right)\left(\sigma_{2}+\sigma_{1}\right)}{2}+\frac{\left(e_{3}-e_{2}\right)\left(\sigma_{3}+\sigma_{2}\right)}{2}+\cdots +\frac{\left(e_{n}-e_{n-1}\right)\left(\sigma_{n}+\sigma_{n-1}\right)}{2}]$$

(2) $$\;\;\;SEA=\frac{W}{\rho th(1-prosity)}$$

In situ compression tests were conducted using a CCD camera to capture real-time deformation of the specimens at various strain levels. Digital image correlation (DIC) analysis was performed in Vic-2D 2009 software (Correlated Solutions, Inc., Irmo, SC, USA) to evaluate the strain distribution during the compression tests. The DIC technique correlates surface images taken before and after deformation, enabling the measurement of local displacements and strains on the specimen surface [40]. It is widely recognized as an effective method for characterizing deformation and failure behavior in various materials [40,41,42]. Therefore, the current study also used the finite element analysis (FEA) Abaqus/Explicit 2020 software to perform quasi-static simulation of the 2D/3D stress concentration distributions of the three porous structures, and a more detailed analysis of the plastic deformation and failure behavior together with the DIC strain distribution results was conducted. This integrated approach facilitated a more comprehensive understanding of the plastic deformation and fracture mechanisms. To enhance computational efficiency and eliminate the influence of strain rate effects, quasi-static simulations were performed using a simplified 5 × 5 × 5 unit cell model instead of the full-scale specimen. This reduced model was sufficient to capture the essential features of the deformation and stress evolution in each lattice architecture, particularly considering that smaller representative volumes allow more accurate predictions of local stress concentrations and deformation patterns [43,44].

## 3. Results

### 3.1. Microstructure of Struts and Relative Density

**Figure 3:**
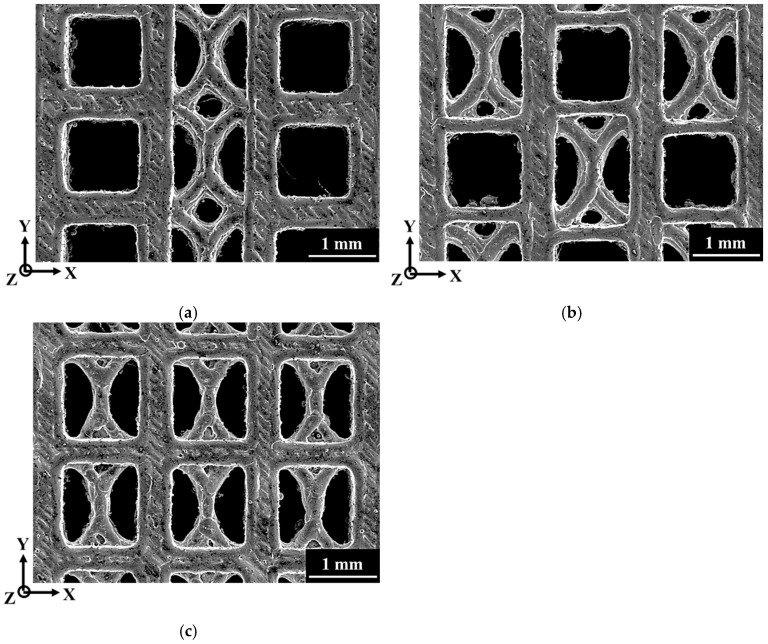
The strut morphologies of Corrax lattice specimens. (**a**) C (**b**) A (**c**) H.

Figure 3 depicts the strut morphologies on the XY plane of the three SLM Corrax lattice specimens (C, A and H). Adherent unmelted powders produced noticeably rough strut surfaces, enlarging the actual strut dimensions relative to the nominal design and thereby altering the measured relative density. Because the unit cell geometries differed, the total surface areas also varied among the specimens. The theoretical surface area and the experimentally determined relative density, calculated in Autodesk Inventor, are summarized in Table 3. Specimen H possessed the largest theoretical surface area, whereas those of specimens C and A were comparable. The measured relative densities were 0.35 ± 0.00153, 0.35 ± 0.00557 and 0.38 ± 0.00058 for C, A and H, respectively. Literature reports [30,45,46] indicate that lattices with larger surface areas tend to retain more unmelted powder during SLM, which explains the higher relative density of specimen H.

### 3.2. Uniaxial Compressive Performance

**Figure 4:**
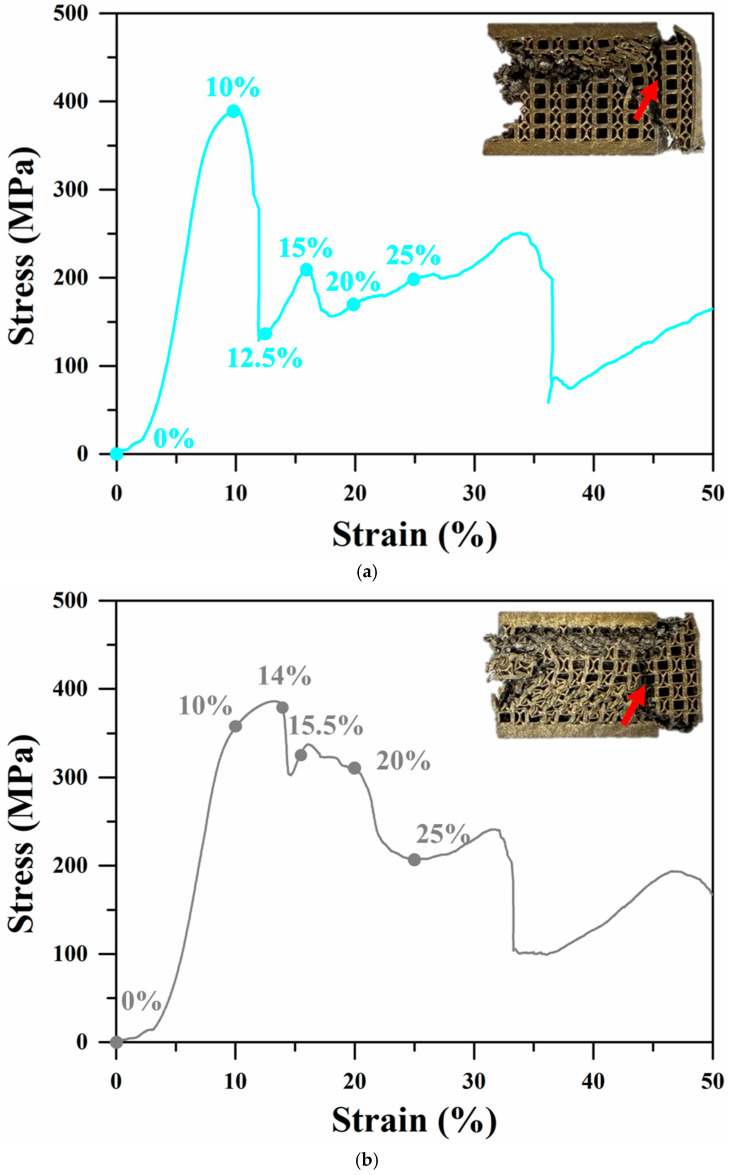

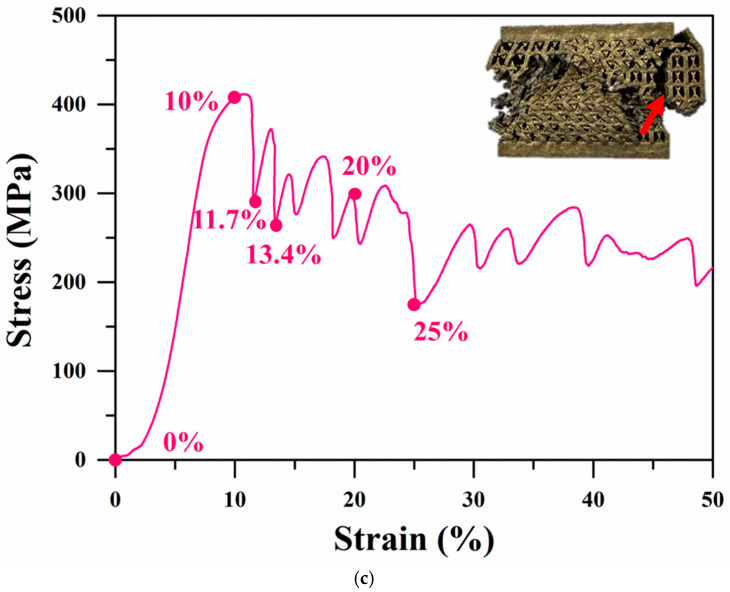
Uniaxial compressive stress–strain curves of (**a**) C (**b**) A (**c**) H specimens after SA treatment.

The uniaxial compressive stress–strain responses and corresponding mechanical properties are presented in Figure 4 and Table 4. All three specimens exhibited a monotonic decrease in stress without a distinct densification plateau, which was consistent with the severe strut fracture observed macroscopically. Specimen H demonstrated the highest first maximum compressive strength (418 ± 5.78 MPa) and energy absorption (128.5 ± 6.83 MJ·m^−3^). Specimens C and A exhibited comparable values for both parameters. Since both first maximum compressive strength and energy absorption scale with relative density [30,45,46], their normalized values—specific strength and specific energy absorption (SEA)—were calculated (Table 4). While the specific strengths showed minimal variation among the specimens, specimen H exhibited the highest SEA, followed by A, with C displaying the lowest value. Despite differences in the overall curve profiles, all lattices exhibited a continuous decrease in compressive stress up to 50% strain. Following the first maximum compressive strength at 10.3% strain, specimen C showed a rapid stress drop (Figure 4a). In specimen A, three notable stress drops occurred at 14%, 20%, and 32% strain (Figure 4b). Specimen H showed recurring minor stress fluctuations, but the overall stress reduction was less severe compared to that of the other two lattices (Figure 4c).

### 3.3. Strain Distribution and Fracture Behavior

The macroscopic morphologies of the specimens after 50% compressive strain, as shown in Figure 4, revealed severe deformation and fracture in all specimens. In particular, prominent outward extrusion of the vertical edge struts was observed, as indicated by the arrows. To further investigate the evolution of deformation and fracture during compression, the strain distribution was analyzed using in situ imaging combined with DIC techniques. However, when the compressive strain exceeded 25%, substantial deformation in the C and H specimens resulted in noticeable mismatches between the measurement plane and the reference image, reducing the accuracy of DIC. Consequently, the DIC analysis was limited to the strain interval between 10% and 25%, where the correlation remained reliable. No significant strain localization was detected at 10% strain, and thus this stage was excluded from further discussion.

**Figure 5:**
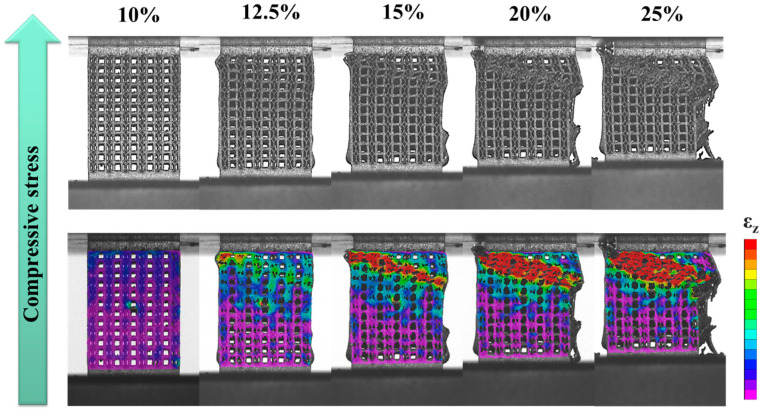
DIC strain maps of C specimen as a function of compressive strain.

**Figure 6:**
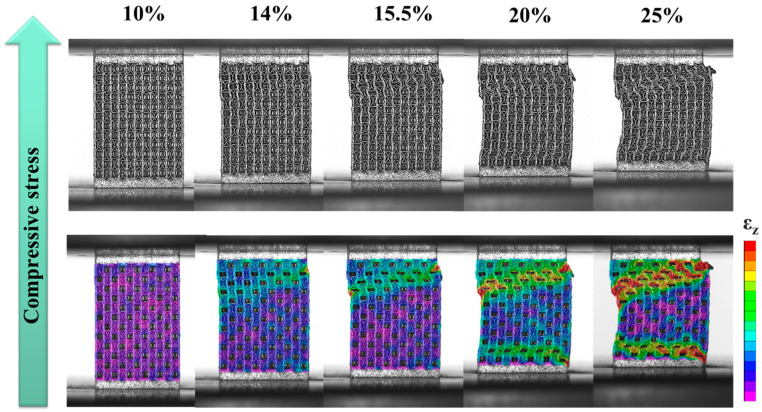
DIC strain maps of A specimen as a function of compressive strain.

**Figure 7:**
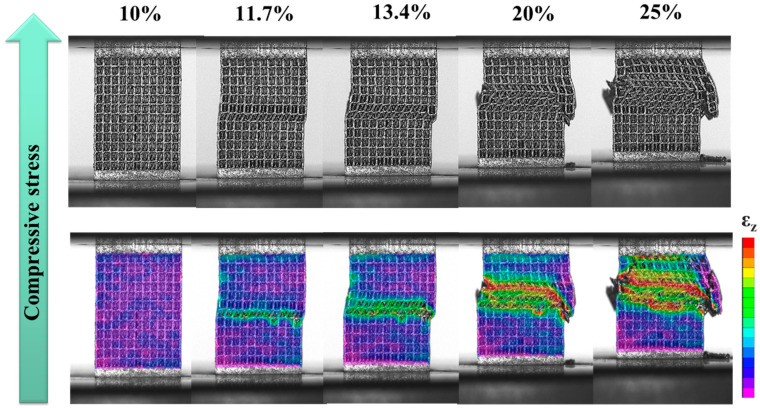
DIC strain maps of H specimen as a function of compressive strain.

Figure 5, Figure 6 and Figure 7 illustrate the evolution of uniaxial compression fracture behavior and the corresponding DIC strain distributions for the three specimens. The color bar indicates the relative strain magnitude, with red and purple representing the highest and lowest strain levels, respectively. As shown in Figure 5, the fracture in the strut of specimen C occurred mainly in the strain concentration zone generated along the compression direction, causing shear deformation and fracture. At 10% strain, it can be observed that local strain concentration began to occur in the FCCZ unit cell at the top of specimen C and a certain middle layer, and the stress dropped rapidly and significantly after the strain reached 10.3%. When the strain reached 12.5%, a shear band at a certain angle to the compression direction could be clearly observed. The FCCZ unit cell around the shear band underwent local severe strain concentration, and a slight strain concentration phenomenon was also observed in the cubic unit cell, causing the top-left corner of the specimen to extrude outward. By 15% strain, the outward extrusion had further intensified, leading to fracture of the vertical struts in the FCCZ unit cell. Additional outward deformation was observed on the far right side of the specimen, accompanied by a drop in stress. As the strain increased, the stress increased slowly while the shear band extended downward. At 20% strain, some vertical struts in the FCCZ unit cell on the right side began to collapse. By 25% strain, the rightmost struts had completely collapsed, making it impossible to use DIC analysis any further. Notably, areas distant from the shear band zone showed no significant deformation during strut collapse.

Figure 6 and Figure 7 show that the fracture in the struts of specimens A and H occurred primarily via layer-by-layer deformation and collapse. As shown in Figure 6, at 14% strain, strain concentration was observed at the node of the cubic unit cell in the upper right corner, as well as in many vertical struts of the cubic unit cells in the upper half, especially at the outermost edges. This led to slight deformation of the vertical struts. At 15.5% strain, the specimen was compressed and deformed from top to bottom, and the cubic unit cells in each layer exhibited more severe deformation compared to the FCCZ unit cells. Outward extrusion was again observed on the far right. Simultaneously, fractures occurred at the nodes of vertical and horizontal struts in both sides of the cubic unit cells. At 20% strain, the strain concentration was primarily in the upper half of the specimen. In addition to severe deformation and fracture in the struts of cubic unit cells, damage was also evident in the struts of FCCZ unit cells. At this time, the bottom of the specimen also began to deform layer by layer. By 25% strain, significant deformation and fracture of the struts had occurred across both upper and lower halves of the specimen, with marked outward extrusion on the right side.

Figure 7 shows that, after the strain reached the first maximum compressive strength (11.07%), a slight stress drop occurred, accompanied by noticeable deformation in the middle layer of the specimen (around 11.7% strain). At 13.4% strain, the stress–strain curve exhibited a secondary minor fluctuation as the deformation extended from the middle layer to adjacent layers. Outward extrusion on the rightmost side and local fracture at the nodes of the vertical strut and the curved strut were observed. With increasing strain, the deformation propagated from the middle layer to the upper region, resulting in progressive collapse. This behavior is consistent with the repeated minor stress fluctuations observed in the stress–strain curve of specimen H (Figure 4c), indicating a relatively stable yet gradual failure process.

### 3.4. Fracture Location and Surface

**Figure 8:**
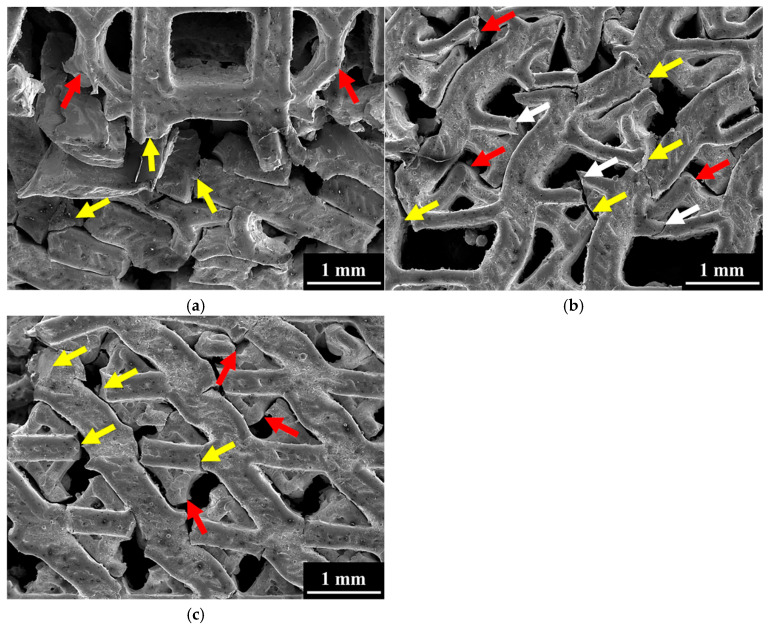
The front view fracture site of (**a**) C (**b**) A (**c**) H specimens under 50% compressive strain.

**Figure 9:**
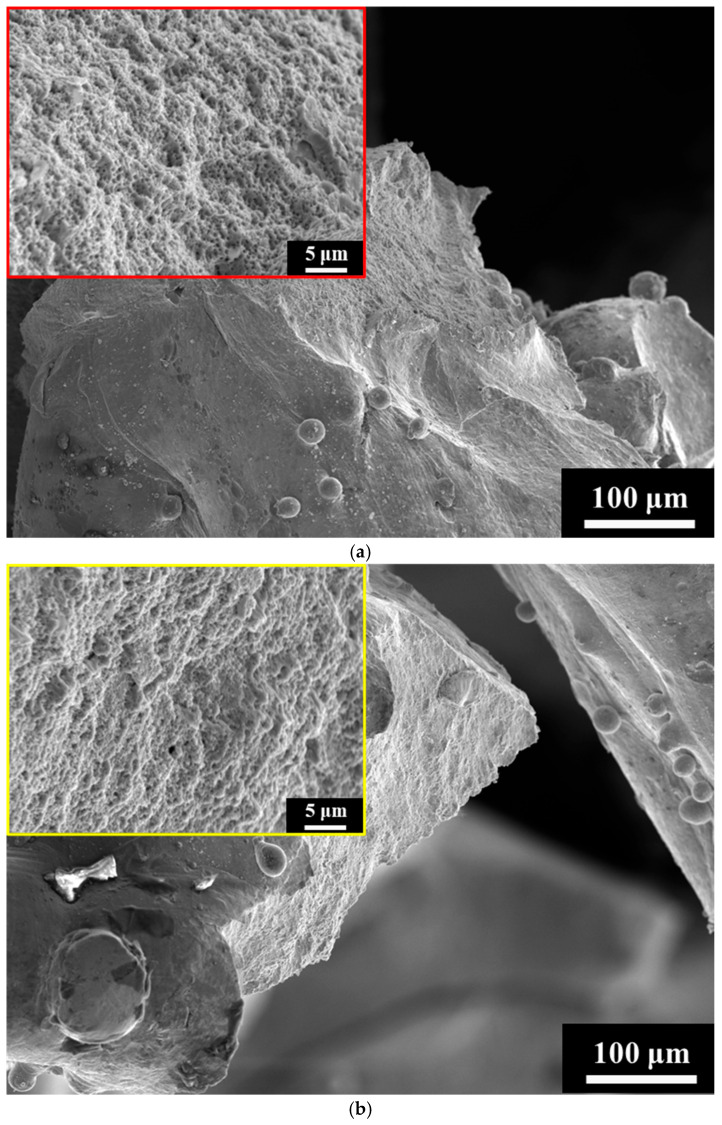

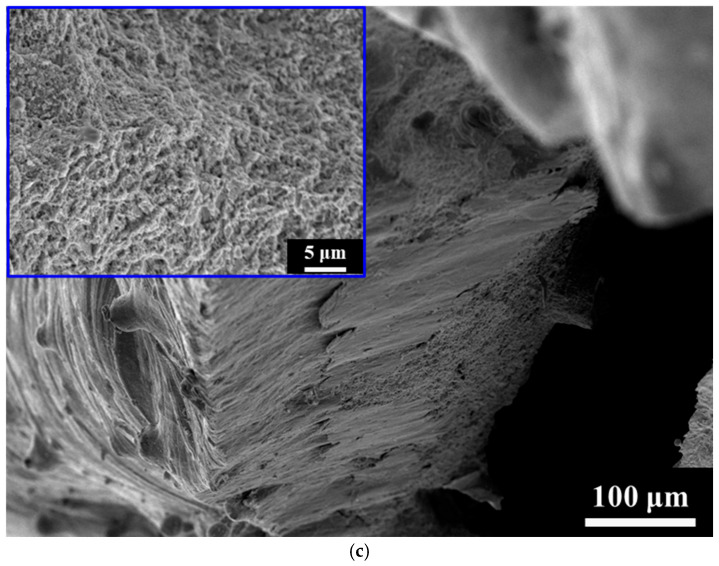
The fracture surfaces of the (**a**) C (**b**) A (**c**) H specimens under 50% compressive strain.

To investigate the locations of the fractures in the struts, SEM was employed to examine the front surfaces of the three specimens after 50% compressive strain, as shown in Figure 8. The fracture sites are indicated by arrows in the figure. The results revealed that, irrespective of unit cell design, most struts experienced severe deformation and fracture. In specimens C and H, the main fracture locations were consistent, primarily occurring at the nodes where vertical and horizontal struts intersected in the cubic unit cell, the nodes in the vertical and curved struts of the FCCZ unit cell (yellow arrows), and the intersections of curved struts in the FCCZ unit cell (red arrows), as shown in Figure 8a,c. In specimen A, similar fracture locations were observed; however, additional fractures were also found along the horizontal struts of the cubic unit cells (white arrows). These fractures were attributed to the fact that the horizontal struts of the cubic unit cells in specimen A were finer than their counterparts in the other two specimens and thus could withstand less stress, as shown in Figure 3 and Figure 8b. Figure 9 presents the fracture surfaces of the three specimens after compression. All specimens exhibited ductile fracture characteristics, as evidenced by the presence of numerous dimples on the fractured strut surfaces. Additionally, smooth regions resembling frictional wear were also observed.

### 3.5. FEA Simulation

**Figure 10:**
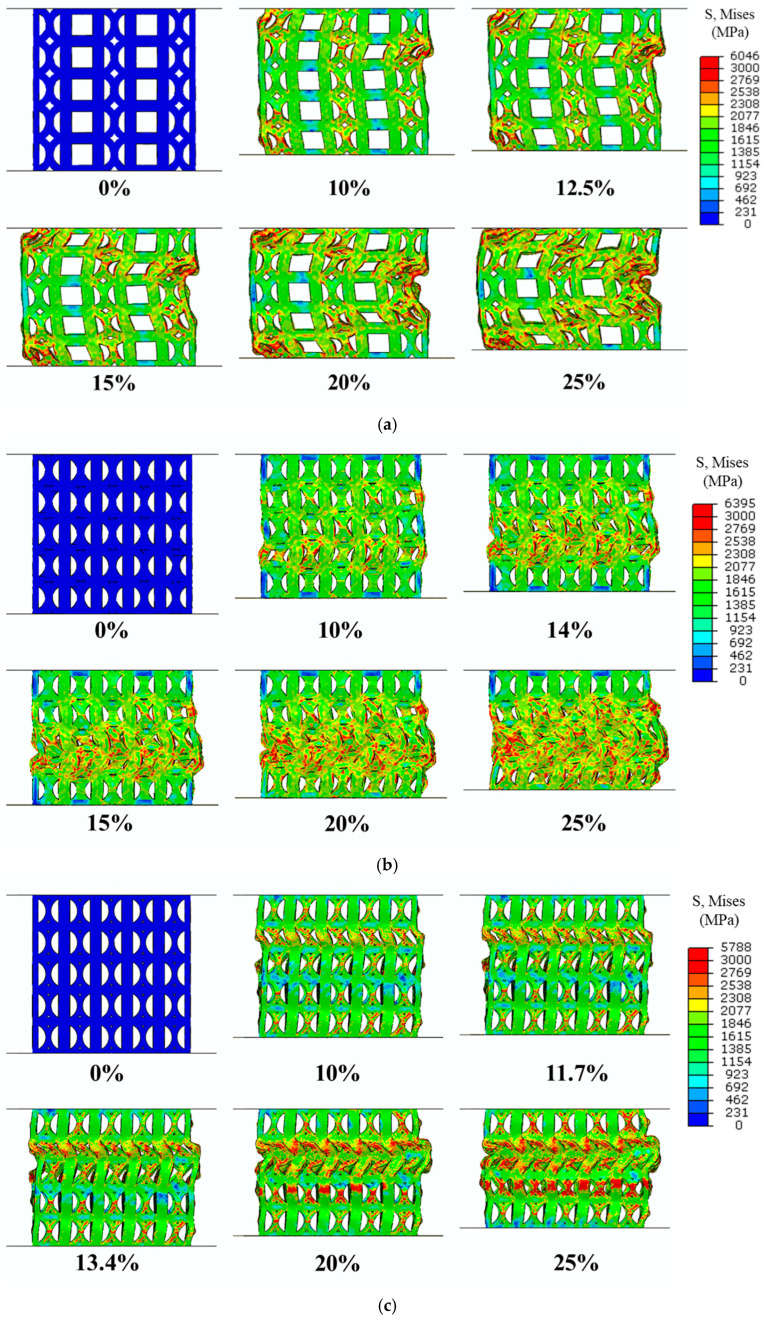
FEA simulation of the stress distributions of (**a**) C (**b**) A (**c**) H specimens from 0% to 25% compressive stress.

Figure 10 shows the deformation and stress distributions of the three specimens during the 0 to 25% compression strain process in the FEA simulation. The specimen compression model consists of three parts from top to bottom: the support plate, the lattice structure, and the loading plate. The color bar on the right represents the von Mises stress on the specimen.

In Figure 10a, when the strain reaches 10%, deformation is observed near the interfaces between the support plates and the loading plate. Specifically, the vertical and curved struts of the FCCZ unit cells, as well as the vertical struts of the cubic unit cells in specimen C, undergo significant deformation. The FCCZ unit cells exhibit more pronounced deformation, with severe buckling observed in the second layer on the far right. Correspondingly, the stress concentrations are also higher in the vertical and curved struts of the FCCZ unit cells, whereas the horizontal struts of the cubic unit cells experience relatively low stress. This indicates that the cubic unit cell in specimen C provides better resistance to compressive loads. As strain increases, substantial deformation extends to the FCCZ unit cells in the central region of the second layer. When the strain reaches 25%, a shear band with a certain angle along the compression direction can be clearly observed, which is similar to the results of DIC analysis in Figure 5.

In specimen A, at 10% strain, initial deformation occurs in the vertical struts of the outermost cubic unit cells. Stress is concentrated at these vertical struts of cubic unit cells and at the intersections, as well as at the curved struts of the adjacent FCCZ unit cells and at the intersections in the same layer. By 14% strain, significant deformation is evident in the second layer near the loading plate and propagates toward the middle layer of cubic unit cells. Buckling of the outer vertical struts of cubic unit cells becomes apparent. With further increases in strain, the middle and surrounding cubic unit cells also undergo severe compression deformation. At 25% strain, the overall structure is nearly flattened, making it difficult to distinguish between the two types of unit cells, as shown in Figure 10b. The FEA simulation confirms that the FCCZ unit cell exhibits strong compressive resistance and that the deformation progresses through a layer-by-layer collapse mechanism, consistent with the experimental observations of specimen A.

In specimen H, at 10% strain, uniform deformation is observed in the second layer near the support plate, with localized buckling in the outermost vertical struts. Stress concentrations are primarily found in the vertical and curved struts of the FCCZ unit cells and at their intersections. As strain increases, deformation continues uniformly through successive layers, reflecting a stable, progressive collapse behavior. This response closely aligns with the experimentally observed deformation behavior of specimen C.

## 4. Discussion

### 4.1. Effect of Lattice Arrangement Design on Compression Fracture Behavior

**Figure 11:**
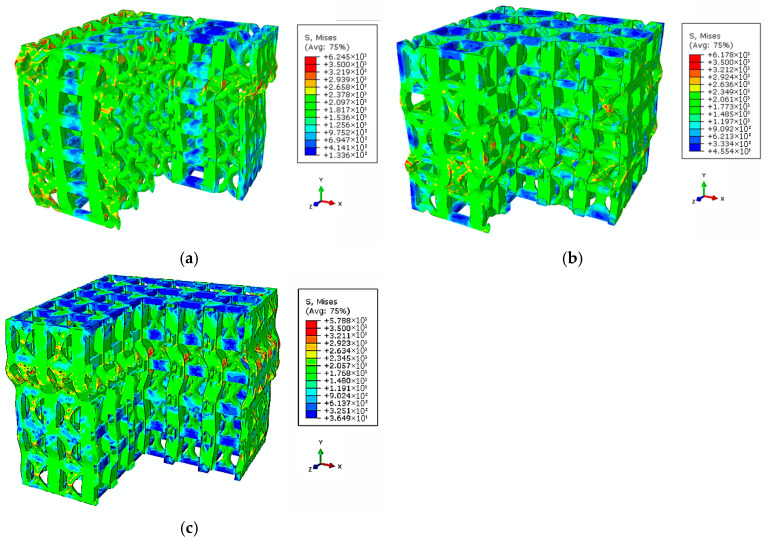
FEA simulation of local stress distributions of (**a**) C (**b**) A (**c**) H specimens under 10% strain.

The compression deformation behaviors of the three specimens were analyzed through both DIC and FEA simulation. The results from the 2D simulations are consistent with the experimental DIC observations, suggesting reliable accuracy in representing deformation patterns [40,42,43]. To assess the internal stress distribution in three dimensions, a 3D FEA simulation was conducted under a compressive strain of 10%, as shown in Figure 11. The simulation revealed that the internal stress distributions in all three specimens were generally consistent with those observed in the 2D FEA. Notably, unit cells containing horizontal struts exhibited lower stress concentrations compared to other regions. Unit cells containing horizontal struts exhibited lower stress concentrations, in agreement with previous studies on hybrid and layered lattice designs [8,29,33].

According to a previous study [30], the FCCZ unit cell is capable of sustaining higher compressive loads than the cubic unit cell. In this study, specimen C and specimen A had cubic-to-FCCZ ratios of 6:5 and 1:1, respectively. Despite specimen C containing more FCCZ unit cells, the overall compressive strengths of specimens C and A were comparable. However, specimen A exhibited a 12.7% higher specific energy absorption (SEA) than specimen C (Table 3), highlighting the influence of unit cell arrangement on load distribution and energy absorption, consistent with previous findings on hybrid and functionally graded lattices [8,30,33].

**Figure 12:**
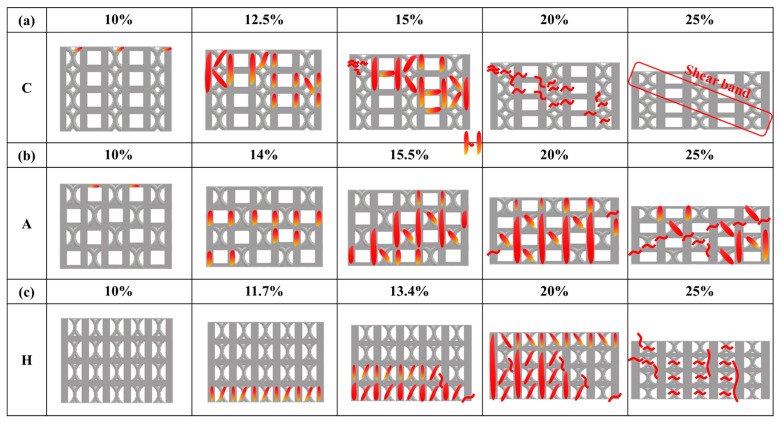
Schematic of the deformation and fracture mechanism of each specimen during uniaxial compression test. (**a**) C (**b**) A (**c**) H specimens. A color change from yellow to red indicates increasing strain concentration severity.

The stress–strain responses further highlight the differences between the two designs. Specimen C experienced a significant stress drop after reaching its first maximum compressive strength, whereas specimen A showed three distinct stress drops at strains of 14%, 20%, and 32%, respectively. DIC and FEA were employed to investigate the deformation and fracture mechanisms in relation to the lattice arrangements and schematic diagrams of each specimen was provided in Figure 12. In specimen C (see Figure 5 and Section 3.3), the fracture was mainly shear deformation and failure along the compression direction. Initial local strain concentrations were observed in FCCZ unit cells near the top and mid-height of the specimen. As the strain increased, a shear band formed at a certain angle to the compression direction, and severe strain concentration and fracture occurred in the FCCZ unit cell. Cubic unit cells also exhibited localized strain concentrations, as shown in Figure 12a. Cubic unit cells in specimen C also exhibited localized strain concentrations. In contrast, specimen A showed layer-by-layer deformation and collapse behavior (see Figure 6 and Section 3.3). Initial strain was concentrated at the node of the cubic unit cell strut located in the upper right corner, especially at the outer vertical struts. As the strain increased, deformation propagated from top to bottom, and the cubic unit cells underwent more severe deformation than the FCCZ units in each layer. Fracture was mainly observed at the nodes of vertical and horizontal struts in the outermost cubic unit cells, as shown in Figure 12b. This load-sharing mechanism delayed failure and increased SEA, consistent with previous reports on hybrid and alternating lattice designs [32,34,47].

From the FEA simulation results of the compressive stress distributions in Figure 10a,b, it can also be verified that the DIC strain distribution results of the two specimens are consistent. When any strain value (10%) was selected, the horizontal strut played a significant role in dispersing stress on the FCCZ unit cell. In specimen A, where cubic and FCCZ unit cells were arranged alternately, the FCCZ units shared horizontal struts with the cubic units, effectively reducing the stress on the vertical struts of FCCZ unit cells (as shown on the outermost side of Figure 10b). The FCCZ unit cell was made to bear the compressive load evenly, and deformation initially occurred at the vertical strut of the cubic unit cell with lower strength. In contrast, the FCCZ unit cells of specimen C lacked horizontal support, concentrating compressive loads on vertical/bent struts and leading to earlier deformation initiation (as shown on the outermost side of Figure 10a). Similar load-sharing mechanisms have been identified in other SLM lattice structures where strut orientation governs stress distribution and fracture patterns [44,45].

Overall, the differences in compressive behavior between specimens C and A are primarily attributed to the arrangements of cubic and FCCZ unit cells. In specimen C, both the cubic and FCCZ unit cells were arranged in a columnar manner. During compression, stress easily accumulated and diffused on the FCCZ unit cells, causing shear fracture of the specimen at a certain angle along in the compression direction [30,48,49]. While the columnar FCCZ design offers high strength [49] in early compression stages, it also promotes rapid crack propagation once local failure initiates. Therefore, after the first maximum compressive strength, the stress drops rapidly and significantly. Conversely, the alternating arrangement in specimen A distributed stress more evenly, delaying structural collapse. Though initial deformation occurred in the cubic units, the surrounding FCCZ cells contributed additional load-bearing capacity, resulting in higher energy absorption and more stable fracture behavior [30]. These observations are supported by prior studies on SLM lattice structures, where unit cell arrangement significantly affected compressive performance, fracture patterns, and SEA [28,29,30,50].

### 4.2. Advantages of Hybrid Lattice Design

As shown in Figure 4 and Table 3, specimen H exhibited superior compressive performance compared to specimens C and A. Its first maximum compressive strength increased by 6.63% and 8.01%, while its SEA improved by 21.76% and 8.07%, respectively. Unlike specimens C and A, specimen H showed repeated stress fluctuations and a gradual decline in compressive strength with increasing strain. This behavior reflects a more stable deformation and energy absorption mechanism [29].

From the strain distributions (Figure 5, Figure 6 and Figure 7) and the analysis in Section 3.3, the deformation and fracture behavior of specimen H closely resembled that of specimen A, with both exhibiting layer-by-layer collapse, as shown in Figure 12c. In contrast, specimen C displayed a distinct shear fracture mode. The difference is attributed to the unit cell design of specimen H, which consisted entirely of hybrid unit cells—each integrating features of both the cubic and FCCZ unit cells. This design combined the mechanical advantages of both geometries, influencing the deformation and fracture behavior [30,47,51].

DIC strain maps (Figure 7) and FEA stress distributions (Figure 10c) show that during compression, stress in specimen H was evenly distributed across the vertical/bent struts of FCCZ unit cells and the vertical struts of cubic unit cells and its nodes. Horizontal struts, however, remained relatively unstressed in both 2D and 3D FEA simulations. Since the stress withstanding by the horizontal strut was relatively small, the H specimen presented layer-by-layer deformation and collapsed during the compression process [8,48]. After the deformation and collapse of each layer, the deformation spread to adjacent layers, creating a stress–strain response with small repetitive fluctuations. The even stress distribution allowed each layer to sustain higher loads, contributing to a slower reduction in compressive strength and superior SEA. Among the three designs, specimen H outperformed specimens C and A in the first maximum compressive strength, energy absorption and SEA. This is attributed to the complex geometry and support method of the hybrid unit cell, which enabled it to withstand higher loads and superior SEA [33,34,49].

Previous studies [33,34] have shown that hybrid architectures can enhance both specific strength and SEA compared to those of single unit cell designs. However, Rahimi et al. [31] reported a hybrid unit cell that improved strength but reduced SEA, underscoring that the performance of hybrid designs is highly sensitive to the chosen unit cell types and their arrangement. In contrast, the hybrid lattice proposed in this study achieves a notable improvement in SEA while maintaining comparable specific strength, thereby validating the effectiveness of the present design strategy.

Based on the findings in Section 4.1, it is evident that the unit cell arrangement plays a more critical role in compressive behavior than the mere presence of high-strength unit cells (e.g., FCCZ). In specimen H, the integration of cubic and FCCZ features within a single hybrid unit cell resulted in a more uniform stress distribution, and improved energy absorption [28,29,30,47]. Therefore, hybrid lattice structures significantly enhance the mechanical performance of SLM Corrax specimens, providing higher compressive strength, superior SEA, and more stable deformation and fracture characteristics. Additionally, incorporating horizontal struts into the lattice design is effective in reducing stress concentrations on vertical struts. When utilizing multiple types of unit cells, an alternating arrangement can help minimize stress accumulation in weaker unit cells and delay the onset of deformation and fracture. This study demonstrates that hybrid and alternated lattice structures are promising strategies for optimizing compressive performance in additive manufactured metallic lattices in the future.

## 5. Conclusions

1. Specimen H, composed entirely of hybrid unit cells integrating cubic and FCCZ features, exhibited the highest first maximum compressive strength (418 ± 5.78 MPa) and specific energy absorption (SEA) (128.5 ± 6.83 MJ·m^−3^), outperforming specimens C and A. The enhanced performance of specimen H is attributed to the combined mechanical advantages of cubic and FCCZ geometries, which facilitated uniform stress distribution, delaying the onset of deformation and layer-by-layer progressive collapse.
2. The unit cell arrangement was found to critically influence the compressive behavior. Alternating or hybrid arrangements reduced stress concentrations on weaker struts and improved energy absorption compared to columnar arrangements, confirming the importance of lattice topology design in structural optimization. DIC and FEA analyses provided detailed insights into deformation propagation and fracture locations, validating the effectiveness of the hybrid design strategy.
3. Hybrid and alternated lattice designs are effective strategies to enhance mechanical performance in additively manufactured metallic lattices. The findings of this study offer practical guidance for the design of lightweight, high-strength, and energy-absorbing components in tooling and structural applications.
4. Future research may explore graded hybrid lattices, the optimization of unit cell geometry for specific loading conditions, and the integration of multi-material additive manufacturing for multifunctional performance enhancement.

## Figures and Tables

**Table 1:** Chemical composition of Corrax stainless steel (wt.%).

C	Si	Mn	Cr	Ni	Mo	Al	Fe
0.033	0.3	0.3	12.0	9.2	1.4	1.6	Bal.

**Table 2:** The process parameters of Corrax stainless steel by SLM.

Laser Power (W)	Scanning Speed (mm/s)	Layer Thickness (μm)	Hatch Spacing (μm)	Layer Rotation (°)
260	1070	100	30	67

**Table 3:** The actual relative densities and surface areas of C, A and H specimens without the top and bottom solid layers.

Lattice Type	Relative Density	Surface Area (mm^2^)
C	0.35 ± 0.00153	26,063.68
A	0.35 ± 0.00557	25,850.9
H	0.38 ± 0.00058	29,622.58

**Table 4:** Compressive performance as a function of lattice type.

Lattice Structure	Relative Density	First Maximum Compressive Strength (MPa)	Energy Absorption (MJ/m^3^)	Specific Strength (kN.m/kg)	SEA (kJ/kg)
C	0.35 ± 0.00153	392 ± 2.41	97.05 ± 7.16	147 ± 0.59	36.3 ± 2.53
A	0.35 ± 0.00557	387 ± 6.92	108.1 ± 6.40	146 ± 3.67	40.9 ± 3.02
H	0.38 ± 0.00058	418 ± 5.78	128.5 ± 6.83	144 ± 1.83	44.2 ± 1.48

## Data Availability

The datasets presented in this article are not readily available because the data are part of an ongoing study.

## References

[1] Soni N., Renna G., Leo P. (2024). Advancements in Metal Processing Additive Technologies: Selective Laser Melting (SLM). Metals.

[2] Hussain A., Kim D. (2025). Fabrication of metal alloy structures with overhang features in laser-based powder bed fusion: A critical review of challenges and latest developments. J. Manuf. Process..

[3] Sanjari M., Mahmoudiniya M., Pirgazi H., Tamimi S., Ghoncheh M.H., Shahriairi A., Hadadzadeh A., Amirkhiz B.S., Purdy M., Araujo E.G.D. (2022). Microstructire, texture, and anistropic mechanical behavior of selective laser melted maraging stainless steel. Mater. Charact..

[4] Bakhtiarian M., Omidvar H., Mashhuriazar A., Sajuri Z., Gur C.H. (2024). The effects of SLM process parameters on the relative density and hardness of austenitic stainless steel 316L. J. Mater. Res. Technol..

[5] Wu M.W., Ku S.W., Yen H.W., Ku M.H., Chang S.H., Ni K., Shih Z.S., Tsai C., Hsu T.W.C., Li L. (2023). The synergic effect of heat treatment and building direction on the microstructure and anisotropic mechanical properties of laser powder bed fusion Corrax maraging stainless steel. Mater. Sci. Eng. A.

[6] Zhou C., Yan X., Wang H., Huang Y., Xue J., Li J., Li X., Han W. (2025). Advancements in hydrogen embrittlement of selective laser melting austenitic stainless steel: Mechanisms, microstructures, and future directions. J. Mater. Sci. Technol..

[7] Garcia-Cabezon C., Castro-Sastre M.A., Fernandez-Abia A.I., Rodriguez-Mendez M.L., Martin-Pedrosa F. (2022). Microstructure-hardness-corrosion performance of 17-4 precipitation hardening stainless steels processed by selective laser melting in comparison with commercial alloy. Met. Mater. Int..

[8] Wu M.W., Ni K., Yen H.W., Chen J.K., Wang P., Tseng Y.J., Tsai M.K., Wang S.H., Lai P.H., Ku M.H. (2023). Revealing the intensified preferred orientation and factors dominating the anisotropic mechanical properties of laser powder bed fusion Ti-6Al-4V alloy after heat treatment. J. Alloys Compd..

[9] Tian Y., Ren H., He J., Zha X., Lin K., Zhou M., Xiong Y. (2024). Surface roughness improvement of Ti-6Al-4V alloy overhang structures via process optimization for laser-powder bed fusion. J. Manuf. Process..

[10] He Y., Zhang F., Dai Y., Zhao K., Ye Z., Yu Z., Xia C., Tan H. (2024). Enhanced low cycle fatigue properties of selective laser melting Ti–6Al–4V with fine-tuned composition and optimized microstructure. J. Mater. Sci. Technol..

[11] Alam M.P., Melaibari A.A., Imam M. (2025). Enhancing surface quality, ductility, and corrosion resistance of SLM fabricated Ti6Al4V alloy through friction stir processing. Mater. Today Commun..

[12] Chang K.C., Zhao J.R., Hung F.Y. (2023). Effects of the microstructure and texture characteristics on mechanical properties of the Al–Ni–Cu–Fe alloy manufactured by laser powder bed fusion. J. Mater. Res. Technol..

[13] Yu S.J., Wang P., Li H.C., Setchi R., Wu M.W., Liu Z.Y., Chen Z.W., Waqar S., Zhang L.C. (2023). Heterogeneous microstructure and mechanical behaviour of Al-8.3Fe-1.3V-1.8Si alloy produced by laser powder bed fusion. Virtual Phys. Prototyp..

[14] Ku M.H., Chuang Y.T., Yu S., Setchi R., Peng J., Wang X., Wang P., Wu W.M. (2025). Superior compressive performance of selective laser melted Al-8.3Fe-1.3V-1.8Si alloy lattice. Mater. Res. Bull..

[15] Yang X.G., Li B., Wang M.L., Guo S.Q., Miao G.L., Shi D.Q., Fan Y.S. (2024). Correlation between microstructures and mechanical properties of a SLM Ni-based superalloy after different post processes. J. Mater. Res. Technol..

[16] Chen B., Zhuo L., Xie Y., Huang S., Wang T., Yan T., Gong X., Wang T., Yan T., Gong X. (2024). Comparative study on microstructure, mechanical and high temperature oxidation resistant behaviors of SLM IN718 superalloy before and after heat treatment. J. Mater. Res. Technol..

[17] Agyapong J., Mateos D., Czekanski A., Boakye-Yiadom S. (2024). Investigation of effects of process parameters on microstructure and fracture toughness of SLM CoCrFeMnNi. J. Alloys Compd..

[18] Mao J., Zhu W., Ye N., Wu Z., Tang J., Zhuo H., Bagliuk G.A. (2025). Effects of raw powder characteristics on microstructure and mechanical properties of W-20Cu composites manufactured by SLM. Int. J. Refract. Met. Hard Mater..

[19] Li P., Ma Y.E., Sun W., Qian X., Zhang W. (2021). Fracture and failure behavior of additive manufactured Ti-6Al-4V lattice structure under compressive load. Eng. Fract. Mech..

[20] Gandhi R., Salmi M., Roy B., Paglari L., Concli F. (2025). Mechanical performance, fatigue behaviour, and biointegration of additively manufactured architected lattices. Virtual Phys. Prototyp..

[21] Zhang X., Jiang L., Yan X., Wang Z., Li X., Fang G. (2023). Regulated multi-scale mechanical performance of functionally graded lattice materials based on multiple bioinspired patterns. Mater. Des..

[22] Zhu G., Xu F., Wang X., Zhang X., Li J. (2025). Mechanical performance of graded lattice structures with periodic density variations fabricated by selective laser melting. Mater. Des..

[23] Kanwar S., Al-Ketan O., Vijayavenkataraman V. (2022). A novel method to design biomimetic, 3D printable stochastic scaffolds with controlled porosity for bone tissue engineering. Mater. Des..

[24] Maskery I., Aboulkhair N.T., Aremu A.O., Tuck C.J., Ashcroft I.A., Wildman R.D. (2016). A mechanical property evaluation of graded density Al-Si10-Mg lattice structures manufactured by selective laser melting. Mater. Sci. Eng. A.

[25] Zhang J., Liu Y., Babamiri B.B., Zhou Y., Dargusch M., Hazeli K., Zhang M.X. (2022). Enhancing specific energy absorption of additively manufactured titanium lattice structures. Addit. Manuf..

[26] Bai L., Xu Y., Chen X., Xin L., Zhang J., Li K., Sun Y. (2021). Improved mechanical properties and energy absorption of Ti-6Al-4V laser powder bed fusion lattice structures using curving lattice struts. Mater. Des..

[27] Zou T., Ou Y. (2025). Nest hybridization of BCC and TPMS lattices A design for high efficiency energy absorption in additive manufacturing. Eng. Struct..

[28] Zhao M., Li X., Yan X., Zhou N., Pang B., Peng B., Zeng Z. (2025). Machine learning accelerated design of lattice metamaterials for customizable energy absorption. Thin-Walled Struct..

[29] Li D., Chen B., Yue D., Sun T., Zhang X. (2024). Hierarchical design and coupling deformation of lattice structures with variable unit cells manufactured by laser powder bed fusion. Thin-Walled Struct..

[30] Wu M.W., Lin Q.E., Ni K., Wang P., Ku M.H., Chang S.H., Chiu J.L., Hsin T.E., Li C.L., Wang C.K. (2024). Novel functionally-graded material design of additive manufactured Corrax maraging stainless steel lattice. Mater. Des..

[31] Rahimi S., Asghari M. (2025). Design and evaluation of two proposed hybrid FCC-BCC lattice structure for enhanced mechanical performance. Heliyon.

[32] Ma X., Zhang N., Tian X. (2024). A novel hybrid lattice structure for improving compression mechanical properties. Mech. Adv. Mater. Struct..

[33] Xiao L., Xu X., Feng G., Li S., Song W., Jiang Z. (2022). Compressive performance and energy absorption of additively manufactured metallic hybrid lattice structures. Int. J. Mech. Sci..

[34] Gharehbaghi H., Farrokhabadi A., Noroozi Z. (2024). Introducing a new hybrid surface strut-based lattice structure with enhanced energy absorption capacity. Mech. Adv. Mater. Struct..

[35] Chang C., Yan X., Deng Z., Lu B., Bolot R., Gardan J., Deng S., Chemkhi M., Liu M., Liao H. (2022). Heat treatment induced microstructural evolution, oxidation behavior and tribological properties of Fe-12Cr-9Ni-2Al steel (CX steel) prepared using selective laser melting. Surf. Coat. Technol..

[36] Zhang J., Wang M., Niu L., Liu J., Wang J., Liu Y., Shi Z. (2021). Effect of process parameters and heat treatment on the properties of stainless steel CX fabricated by selective laser melting. J. Alloys Compd..

[37] Wu M.W., Chen J.K., Tsai M.K., Wang P., Cheng T.L., Lin B.H., Chiang P.H., Dhinakar A. (2022). Uniaxial compression properties and compression fatigue performance of selective laser melted Ti-6Al-4V cellular structure. Met. Mater. Int..

[38] Yang J., Liu H., Cai G., Jin H. (2025). Additive Manufacturing and Influencing Factors of Lattice Structures: A Review. Materials.

[39] (2011). Mechanical Testing of Metals-Ductility Testing Compression Test for Porous and Cellular Metals.

[40] Chu T.C., Ranson W.F., Sutton M.A. (1985). Applications of digital-image-correlation techniques to experimental mechanics. Exp. Mech..

[41] Brenne F., Niendorf T. (2019). Load distribution and damage evolution in bending and stretch dominated Ti-6Al-4V cellular structures processed by selective laser melting. Int. J. Fatigue.

[42] Popławski A., Bogusz P., Grudnik M. (2025). Digital Image Correlation and Numerical Analysis of Mechanical Behavior in Photopolymer Resin Lattice Structures. Materials.

[43] Huang Z., Antion C., Toussaint F. (2025). Identification for elastoplastic constitutive parameters of 316L stainless steel lattice structures using finite element model updating and integrated digital image correlation. Mech. Mater..

[44] Zhang L., Wu J., Zhao Y., Qin Z. (2025). Mechanical behavior and energy absorption capability of trigonometric function curved rod cell-based lattice structures under compressive loading. Sci. Rep..

[45] Teng F., Sun Y., Guo S., Gao B., Yu G. (2022). Topological and Mechanical Properties of Different Lattice Structures Based on Additive Manufacturing. Micromachines.

[46] Choy S.Y., Sun C.N., Leong K.F., Wei J. (2017). Compressive properties of Ti-6Al-4V lattice structures fabricated by selective laser melting: Design, orientation and density. Addit. Manuf..

[47] Li N., Pang S., Chen S., Liu Y., Aiyiti W., Chen Z. (2024). Design and application of hybrid lattice metamaterial structures with high energy absorption and compressive resistance. J. Mater. Res. Technol..

[48] Yang Y., Wang S., Yan S., Zhao Y., Zhang Y. (2025). Enhancing the mechanical performance of additively manufactured lattice structure via locally reinforcing struts: Coupled influence of structure and microstructure. Mater. Sci. Eng. A.

[49] Lin Q.E., Wu C.D., Zhang Y.W., Li C.L., Ku M.H., Chang S.H., Wu M.W. (2024). Enhancement of the compressive performances of additive manufactured Corrax maraging stainless steel lattice by heat treatment. J. Mater. Res. Technol..

[50] Park K.M., Roh Y.S., Lee B.C. (2024). Effects of the unit-cell size and arrangement on the compressive behaviors of lattice structures in powder bed fusion additive manufacturing. Results Mater..

[51] Emir E., Bahçe E. (2025). Compressive behavior of functional graded hybrid lattice structure. Int. J. Adv. Manuf. Technol..

